# Simulation of GHz ultrasonic wave piezoelectric instrumentation for Fourier transform computation

**DOI:** 10.1038/s41598-023-42191-1

**Published:** 2023-09-12

**Authors:** Zaifeng Yang, Xing Haw Marvin Tan, Viet Phuong Bui, Ching Eng Png

**Affiliations:** Institute of High Performance Computing (IHPC), Agency for Science, Technology and Research (A*STAR), 1 Fusionopolis Way, Connexis #16-16, Singapore, 138632 Republic of Singapore

**Keywords:** Electrical and electronic engineering, Mechanical engineering, Acoustics

## Abstract

The recent emerging alternative to classic numerical Fast Fourier transform (FFT) computation, based on GHz ultrasonic waves generated from and detected by piezoelectric transducers for wavefront computing (WFC), is more efficient and energy-saving. In this paper, we present comprehensive studies on the modeling and simulation methods for ultrasonic WFC computation. We validate the design of the WFC system using ray-tracing, Fresnel diffraction (FD), and the full-wave finite element method (FEM). To effectively simulate the WFC system for inputs of 1-D signals and 2-D images, we verified the design parameters and focal length of an ideal plano-concave lens using the ray-tracing method. We also compared the analytical FFT solution with our Fourier transform (FT) results from 3-D and 2-D FD and novel 2-D full wave FEM simulations of a multi-level Fresnel lens with 1-D signals and 2-D images as inputs. Unlike the previously reported WFC system which catered only for 2-D images, our proposed method also can solve the 1-D FFT effectively. We validate our proposed 2-D full wave FEM simulation method by comparing our results with the theoretical FFT and Fresnel diffraction method. The FFT results from FD and FEM agree well with the digitally computed FFT, with computational complexity reduced from $O(N^2 logN)$ to *O*(*N*) for 2-D FFT, and from *O*(*NlogN*) to *O*(*N*) for 1-D FFT with a large number of signal sampling points *N*.

## Introduction

Fourier transform (FT) is commonly used in a wide variety of digital computations^1^, including signal and image processing, solving differential equations, artificial intelligence (AI) models, etc. Repetitive FFT computations could lead to considerable power consumption and prevent real-time signal/image processing, especially when the dimension of the input data is extremely large. For example, many types of image processing are implemented in frequency/spectral domain such as de-noising, edge detection, etc. Thus, FFT could transform the image from spatial domain to the corresponding frequency counterpart^2^. Image processing techniques have flourished in the recent years with the rapid development of deep learning methods, especially for those based on convolutional neural networks (CNN)^3^. Repetitive CNN calculation is needed for training or running a trained model with various inputs. In this case, FFT also can be used for convolutional calculation, given that the convolution of two images is equivalent to the multiplication of the FFT results of the two images. For example, FFT acceleration using photonics for AI is an ongoing heated topic^4^, and photonic integrated circuits can do the FFT physically rather than digitally^5,6^. The 2-D FFT for an image with $N \times N$ pixels has a computational complexity of $O(N^2 logN)$. Obviously, the computational complexity would be exponentially increased if the number *N* (the size of the image) becomes larger. Unlike 2-D image processing, signal processing^7^ based on electromagnetic waves is usually based on 1-D temporal input data. Usually, real-time frequency response is required for applications such as object detection, recognition, distance measurement, etc. In these scenarios where repetitive FFT computations are usually needed, excessive energy will be consumed and the efficiency could be also low if the resolution of the input signal is high (large 1-D input data). Additionally, the computational complexity of the 1-D FFT for signals is *O*(*NlogN*). Similar to the 2-D FFT computation, the computational complexity would become high if the number *N* (the sampling points of the signal) is large.

Instead of calculating the FFT digitally by computer using the Cooley–Tukey algorithm^8^, there are some alternative methods to implement FFT physically. At the Fourier plane of a 4*f* optical lens system^9,10^, the diffraction pattern shows the Fourier transformation of the input 2-D image, where low frequency components are located close to the optical axis and higher frequency ones are placed farther away from the origin. Photonic integrated circuits (PIC)^5,6^ are another efficient method to achieve Fourier transform. By choosing the angular locations of the input and output waveguides, the star coupler can implement a discrete Fourier transform. However, the resolution of the Fourier transform result is not high and such a component is difficult to be integrated with the other electronic devices.

Recently, an emerging ultrasonic wavefront computing (WFC) technique was proposed to compute the FFT^11,12^. This method uses the principles of wave mechanics in the acoustic domain by implementing the Fourier transform through ultrasonic waves propagating within Silicon. According to Patel et al.^12^, the computational complexity of WFC is $O(\delta )$, where $\delta$ is the transit time of the ultrasonic wavefront. This is because the number of cycles consumed in the microprocessor is comparable to the transit time of the ultrasonic wavefront. As a result, WFC can achieve a significant speedup over CPU-computed FFT algorithms. For a WFC module with an $N \times N$ transducer array, the computational complexity is *O*(*N*). The WFC technique achieves a $2317\times$ system-level energy-delay product and benefits a simultaneous 117.69$\times$ speedup with 19.69$\times$ energy reduction, as compared to the state-of-the-art baseline all-digital configuration^12^. Table 1 summarizes the above mentioned physical Fourier transform realization approaches against digital computation, in terms of the complexity and its pros and cons.

**Table 1:** A summary of some physical Fourier transform realization approaches.

Approach	Pros and Cons	Complexity
Cooley–Tukey^8^	Easy to implement with digital signals Computational costly for large domain accuate computationa easy to be implemented in digital equipment	2D: $O(N^2logN)$ 1D: *O*(*NlogN*)
4*f* free-space Lens^9^	Extremely fast High resolution Difficult to integrate with electronic devices Difficult to obtain the phase Delicate experimental setup	2D: *O*(*N*)
Photonic integrated circuit (PIC): Star Coupler^5,6^	Highly efficient Low resolution Difficult to integrate with electronic devices	2D: *O*(*N*) 1D: *O*(*N*)
Wavefront computing using GHz ultrasonic piezoelectric transducers^11,13^	Highly efficient Easy to fabricate Integrated with the other electronic circuits Energy saving Low cost	2D: *O*(*N*) 1D: *O*(*N*)

To date, the ultrasonic WFC has been investigated only for the FFT of 2D data for image processing, however, 1-D FFT for signal processing is important and how to use such a WFC system for signal processing remains unknown. On the other hand, the WFC system using GHz ultrasonic waves^11,13,14^ passing through a flat lens^15,16^ will be finally packaged into a chip by semiconductor technologies. Before fabrication and measurement, it is important to validate the idea, investigate the main factors that which cause errors, and optimize the system design. To this end, accurate modeling and simulation for such an ultrasonic WFC system is required. However, most verification based on modeling and simulation for such whole system is based on the Fresnel diffraction method without considering the complex material factors such as losses and piezoelectric effects from the transducers^17^.

The contributions of our work are:

- We simulate an emerging GHz ultrasonic wave piezoelectric instrument for computing Fourier transforms. The techniques include ray tracing, Fresnel diffraction and full-wave finite element method (FEM). These methods are used for different tasks: ray tracing simulation can be used to validate the design parameters of the WFC system; Fresnel diffraction simulation is efficient and can handle a larger computational domain; full-wave FEM simulation is the most accurate but it is computationally intensive.
- To the best of our knowledge, we are the first to implement the full-wave FEM simulation for the emerging ultrasonic wavefront computing instrument. Unlike ray-tracing and Fresnel diffraction methods which consider input signals or images based on the transducer shapes, full-wave FEM simulation takes the piezoelectric effects, losses due to the transducers and acoustic blocks, and anisotropic properties of the lens into account. Through full-wave simulation, we can have an insightfully predict the performance of the WFC system to be fabricated, by considering practical factors which will be present in the experiment.
- We perform novel full-wave modeling and simulation methods for both 1-D signals and 2-D images as the input for FFT. Unlike previous reported WFC system which only cater to 2-D images^11,13^, our proposed method also can solve the 1-D FFT effectively. We demonstrate multiple simulation examples which validate our proposed simulation method, by comparing our full-wave FEM simulation results with the results from theoretical FFT and Fresnel diffraction techniques. The computational complexity is reduced from $O(N^2 logN)$ to *O*(*N*) for 2-D FFT, and from *O*(*NlogN*) to *O*(*N*) for 1-D FFT with larger number of *N*.

## Theory

**Figure 1:**
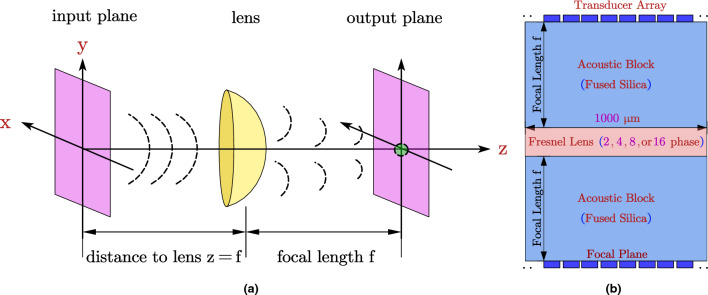
The schematic of the WFC system in (**a**) free space (**b**) solids.

Ultrasonic Fourier transform with a lens only works if the Fresnel approximation or the paraxial approximation is adhered to. In contrast, if a lens is not used, the Fraunhofer approximation is necessary, which is valid only in the Fraunhofer far-field zone, as illustrated in Fig. 1. The paraxial approximation assumes that the waves emitted from the pixels and aperture of the source plane do not diverge at large angles off the normal^9^. In order for this condition to be met, the aperture size must be significantly larger than the wavelength, but much smaller than the path length. The resonant frequency of an aluminium nitride (AlN) piezoelectric transducer, which is a crucial component of ultrasonic FT instrument, is determined by its thickness and other material properties such as its density, elasticity and compliance matrix components. The resonant frequency is the frequency at which the transducer vibrates most efficiently and produces the highest amplitude of acoustic waves. In this context, a 2 $\upmu$m thick AlN piezoelectric transducer results in a resonant frequency between 1.6 and 1.8 GHz, which includes our targeted resonant frequency of 1.7 GHz. The speed of sound in fused silica (SiO$_2$) determines the ultrasonic wavelength, which is the distance between two consecutive peaks or troughs of the wave. In the present scenario, the speed of sound in Fused Silica is c = 5900 m/s, which corresponds to an ultrasonic wavelength of 3.5 $\upmu$m. Thus, an understanding of these important parameters is crucial for the design and implementation of the ultrasonic FT instrument.

To validate the WFC system using the paraxial approximation, the physical size of the pixel $\Delta$ should be much larger than the wavelength: $\Delta \ge 10\lambda$. Here, we choose the pixel size to be 50 $\upmu$m $\times$ 50 $\upmu$m, which is compatible with 130 nm complementary metal-oxide-semiconductor (CMOS) technology. Accordingly, each pixel in the transmitting and receiving piezoelectric arrays has a width and length of $\Delta = \Gamma \lambda$, where $\Gamma \approx 14$. A larger value of ${\Gamma }$ causes the aperture to produce an effectively paraxial wavefront. This consists of a 40 $\upmu$m $\times$ 40 $\upmu$m AlN transducer area with a 10 $\upmu$m gap surrounding every pixel to minimize the acoustic coupling between neighboring pixels. The receiving piezoelectric sensor array also comprises a 50 $\upmu$m $\times$ 50 $\upmu$m array of pixels.

The width of the entire aperture of the transmitting piezoelectric actuator array (along the lateral dimension) is

1 $$\begin{aligned} L_w = {\Delta \times N}={\Gamma N\lambda } \end{aligned}$$

where N refers to the number of pixels, which also corresponds to the number of elements in the transducer array. The length $L_{th}$ which the wave has to travel over can be determined from both Fresnel diffraction approximation, and the requirement for the distance to be sufficiently long to satisfy the constant phase condition (i.e. the sampling condition of the phase term)^9^:

2 $$\begin{aligned} \frac{L_w}{L_{th}}\times \frac{L_w}{4\lambda }\gg 1 \end{aligned}$$

Therefore, $L_{th} \approx \kappa N\lambda$, where $\kappa$ depends on the phasing used in the transmitting piezoelectric actuator transducers, the type of lens used, and also the size of the pixels. Thus, the propagation length $L_{th}$ is effectively linear with respect to the number of array elements *N*^9^. The time taken for the ultrasonic wave to propagate in the medium from the input plane to the output plane (Fig. 1), $t_{transit}$, can be derived as:

3 $$\begin{aligned} t_{transit}=\frac{L_{th}}{c}=\frac{\kappa N\lambda }{c}=\frac{\kappa N}{f}=\kappa NT \end{aligned}$$

where $T=\frac{1}{f} = 0.59$ ns is the period of the ultrasonic wave. The ultrasonic FT system described in this study utilizes a thin lens, thus allowing the omission of its thickness in calculations. At the input plane, waves from each pixel of the transmitting piezoelectric actuator array propagate to the receiving piezoelectric sensor array located at the output plane, taking T cycles to traverse the system. Notably, the ultrasonic frequency of several GHz is comparable to modern micro-processor clock frequencies. This warrants a comparison of the number of cycles required for computation in the micro-processor and the transit time of the ultrasonic wavefront. Computation complexity can be approximated as *O*(*N*), where N represents the number of cycles required by the GHz clock for completion. Consequently, the latency of the FT computation using the wavefront computing (WFC) approach is primarily influenced by the time taken for the wave to travel in the substrate, which is inversely proportional to the speed of sound in the acoustic blocks.

The computation of ultrasonic FT involves summing up k-vectors while adhering to the principles of acoustic wave propagation. To prevent energy loss at the sides, the size of the lens must exceed that of the input aperture $L_w$. This ensures that all rays emitted by the transmitting piezoelectric actuator array are captured. Specifically, the lens must be larger than the covering maximum aperture, which includes the size of the aperture as well as the side gap, and is greater than the spread $\partial D$:

4 $$\begin{aligned} \partial D=\ f\times (\lambda /{ \Delta }) \end{aligned}$$

In conventional lenses, a spherical surface transforms an incident plane wave to a spherical wave, resulting in an emerging spherical wave which is then focused on the focal plane. This satisfies the paraxial approximation^9^. To achieve a thin, compact, and CMOS-compatible WFC instrument, we designed a multi-phase Fresnel lens with a thickness in the range of 20–25 $\upmu$m. This Fresnel lens effects a parabolic phase shift as a function of radius, causing the focusing of ultrasonic waves to produce a Fourier transform at the output plane. It is worth noting that the mechanism is based on diffraction instead of refraction or reflection. The incident waves diffract around the lens and the diffracted waves interfere constructively at the designed focal length^13,18^. All design parameters are listed in Table 2.

**Table 2:** Design parameters and the radius of the curvature in ideal lens, derived from Fresnel optics, for different transducer arrays.

N by N array	4 by 4	8 by 8	32 by 32	128 by 128
Min aperture ($\lambda$) (mm)	0.05	0.05	0.05	0.05
Max aperture (A) (mm)	0.2	0.4	1.6	6.4
Focal length (f) (mm)	0.36	0.72	2.86	11.43
Full length (L = 2f) (mm)	0.71	1.43	5.71	22.86
Radius of the curvature in ideal lens^25^ (mm)	0.12141	0.24283	0.96456	3.85486

## Methods

### Ray-tracing

Ray-tracing is a geometrical optics method used to simulate the behavior of light in optical systems, and we apply it for the ultrasonic WFC system to validate the design parameters such as focal length. It makes use of the Fresnel equations by defining the positions and directions of the input rays. Ray-tracing is used to simulate the behavior of light in optical systems by tracing the path of light rays as they interact with objects in the system. The rays interact with certain modeled objects (e.g. the lens in the WFC system). The directions of the rays are changed due to the refractive index at the interface. The basic idea behind ray-tracing is to model light as a series of rays that originate from a light source and travel through the optical system, interacting with objects along the way. We applied the ray-tracing to acoustic waves. However, the characteristic of how the acoustics waves interact with the complicated medium was not considered. Ray tracing only considers the interaction at the boundary of different materials. Thus, the ray-tracing method cannot obtain the FT results at the focal plane. We used an in-house MATLAB code to implement the ray-tracing method, to validate the focal length design of the WFC system. Our Ray Tracing code in MATLAB based on the equations in Ref.^19^.

### Fresnel diffraction

Fresnel diffraction originated from the field of optics^9^, but has been applied to acoustics^20,21^. The Fresnel approximation is derived from the Huygens–Fresnel Principle^9^. The basic idea behind Fresnel diffraction is that the wavefronts are divided into many small segments, each of which acts as a point source of light. The light from each of these point sources interferes with the light from all of the other point sources to create a diffraction pattern. Thus, it allows us to compute the acoustic pressure field as a complex number after propagating though a medium. Previous works have computed the propagation of acoustic pressure field in air or water^22^. Fresnel diffraction provides a most effective way to model the diffraction of waves. We used the code available from Ref.^23^ to perform our Fresnel diffraction simulations. However, the propagation along the acoustic blocks are simplified. In particular, the piezoelectric effects, losses due to the transducers and acoustic blocks, and anisotropic properties of the lens, cannot be captured by the Fresnel diffraction model.

### Full-wave FEM modeling

In FEM, a complex system is divided into smaller, simpler parts, or “elements”, that can be modeled mathematically. These elements are then connected to form a finite element model of the entire system. The behavior of each element can be described using mathematical equations, and the behavior of the entire system can be calculated by solving these equations. As long as the modeling for FEM is well discretized, the simulation results are usually of high accuracy. We used the software COMSOL Multiphysics for FEM simulations. Perfectly matched layers were used in frequency domain to absorb the waves at the boundaries of the simulation domain. The full-wave FEM method^24^ yields the most accurate results among the three simulation methods due to it takes into account the wave nature (elastic wave for the WFC system) and allows for more accurate modeling of complex optical phenomena (multi-level Fresnel lens), such as diffraction and scattering. The limitation of FEM is it’s computationally intensive nature which requires a lot of computational resources. For this reason, we performed the FEM simulations in 2D. Due to our lack of computational resources, we were unable to perform the FEM simulations in 3D. Other full-wave potential alternatives such as Finite-difference Time-domain (FDTD) or Finite-element Time-domain (FETD) method are even more computationally inefficient. Moreover, the FFT results cannot be directly observed in time-domain simulation.

### Relationship between Fresnel diffraction and theoretical fourier transform

The wavefront computation (WFC) can be modeled using the Fresnel diffraction equation for wavefront propagation. To model the entire wavefront propagation from the transmitters’ pixels to the receivers’ pixels, two Fresnel propagation steps are needed. The first Fresnel propagation step of the complex pressure field is from the transmitters’ pixels (also called the input plane in Fig. 1a) to just in front of the lens. Then, the pressure field needs to be phase transformed by the lens. After phase transformation by the lens, the pressure field needs to be propagated again using Fresnel diffraction to the receivers’ pixels (also called the output plane in Fig. 1a).

From equations (5–19) in Joseph Goodman’s textbook^9^ which are used to perform Fresnel diffraction propagation via computing the Fourier transform, for the special case where *d* = *f*, where *d* is the distance from the object plane to the lens, and *f* is the focal length, then the equation reduces an exact Fourier transform:

5 $$\begin{aligned} U_f\left( x,y\right) =\iint {U_l\left( \xi ,\eta \right) \ exp\left[ -j\frac{2\pi }{\lambda f}\left( \xi x+\eta y\right) \right] \ d\xi \ d\eta } \end{aligned}$$

where $U_l\left( \xi ,\eta \right)$ is the pressure field in the input plane, and $U_f\left( x,y\right)$ is the pressure field in the output plane.

Performing the substitutions $u=\frac{x}{\lambda f}$ and $v=\frac{y}{\lambda f}$ , we get:

6 $$\begin{aligned} F(u,v)=\iint {U_l\left( \xi ,\eta \right) exp\left( -j2\pi \left( u\xi +v\eta \right) \right) \ d\xi d\eta } \end{aligned}$$

The resulting 2-D Fourier transform *F*(*u*, *v*) has frequency axes in the SI units of 1/m. In order to convert the frequency axes to the SI units of meters, we need to multiply the Fourier axes by $\lambda f$, where $\lambda$ is the wavelength, and f is the focal length. This will give us the pressure field $U_f\left( x,y\right)$ in the image plane with spatial axes in SI units of meters.

## Results

### Ray tracing simulation

**Figure 2:**
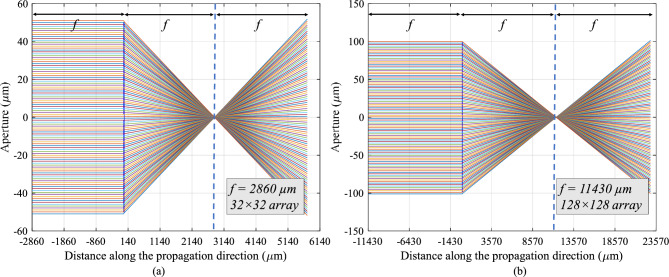
Ray-tracing simulation results. (**a**) 32 $\times$ 32 array with focal length of 2860 $\upmu$m aperture 100 $\upmu$m, 100 rays. (**b**) 128 $\times$ 128 array with focal length of 11,430 $\upmu$m aperture 200 $\upmu$m, 100 rays.

Our ray-tracing simulation^19^ results confirm a focal length of 2.86 mm for the the 32 by 32 transducer array (Fig. 2), consistent with the calculated focal length in Table 2. The focal length of 11.43 mm predicted by Table 2 for the 128 by 128 transducer array was also validated by ray tracing (Fig. 2).

### Full-wave FEM simulations and Fresnel diffraction

**Figure 3:**
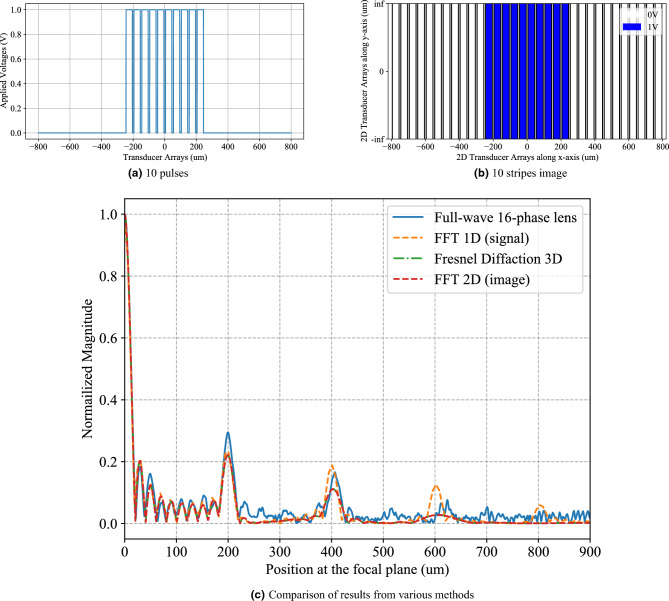
Case 1 which corresponds to the array of 32 piezoelectric transducers with 10 transducers being excited by (**a**) 1-D voltage signals with 10 rectangular signals. (**b**) Image of the input voltages at the input plane having 10 stripes represented by blue and white pixels. (**c**) Validation of the WFC system simulated by the full-wave FEM method, compared with digital FFT and Fresnel diffraction results using 16-phase Fresnel lens.

The 1D voltage signals in Figs. 3a, 6a and 7a were numerically Fourier transformed using the Fast fourier transform package in Python (SciPy). The frequency axes of the numerically Fourier transformed results were converted to the spatial axes as explained in under the Method subsection “Relationship between Fresnel diffraction and theoretical fourier transform”. After conversion to spatial axes, the numerically Fourier transformed results are plotted as “FFT1D(signal)” in Figs. 3c, 6c and 7c. Similarly, the 2D voltage signals in Figs. 3b, 6b and 7b were numerically Fourier transformed using the Fast Fourier transform package in Python (SciPy). The frequency axes of the numerically Fourier transformed results were converted to the spatial axes as explained in under the Method subsection “Relationship between Fresnel diffraction and theoretical fourier transform3.4”. After conversion to spatial axes, the numerically Fourier transformed results are plotted as “FFT2D(image)” in Figs. 3c, 6c and 7c.

**Figure 4:**
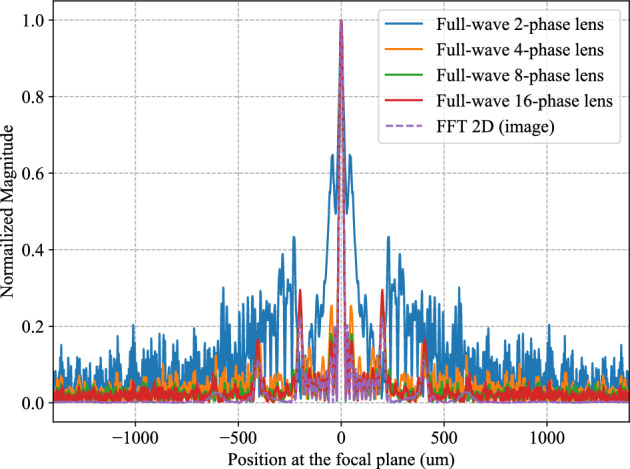
The simulated Fourier transform results for Case 1 which corresponds to the array of 10 piezoelectric transducers excited by 1-D voltage signals with 10 rectangular pulses, using multi-level Fresnel lenses from 2 to 16 phases, compared with theoretical 2-D FFT.

**Figure 5:**
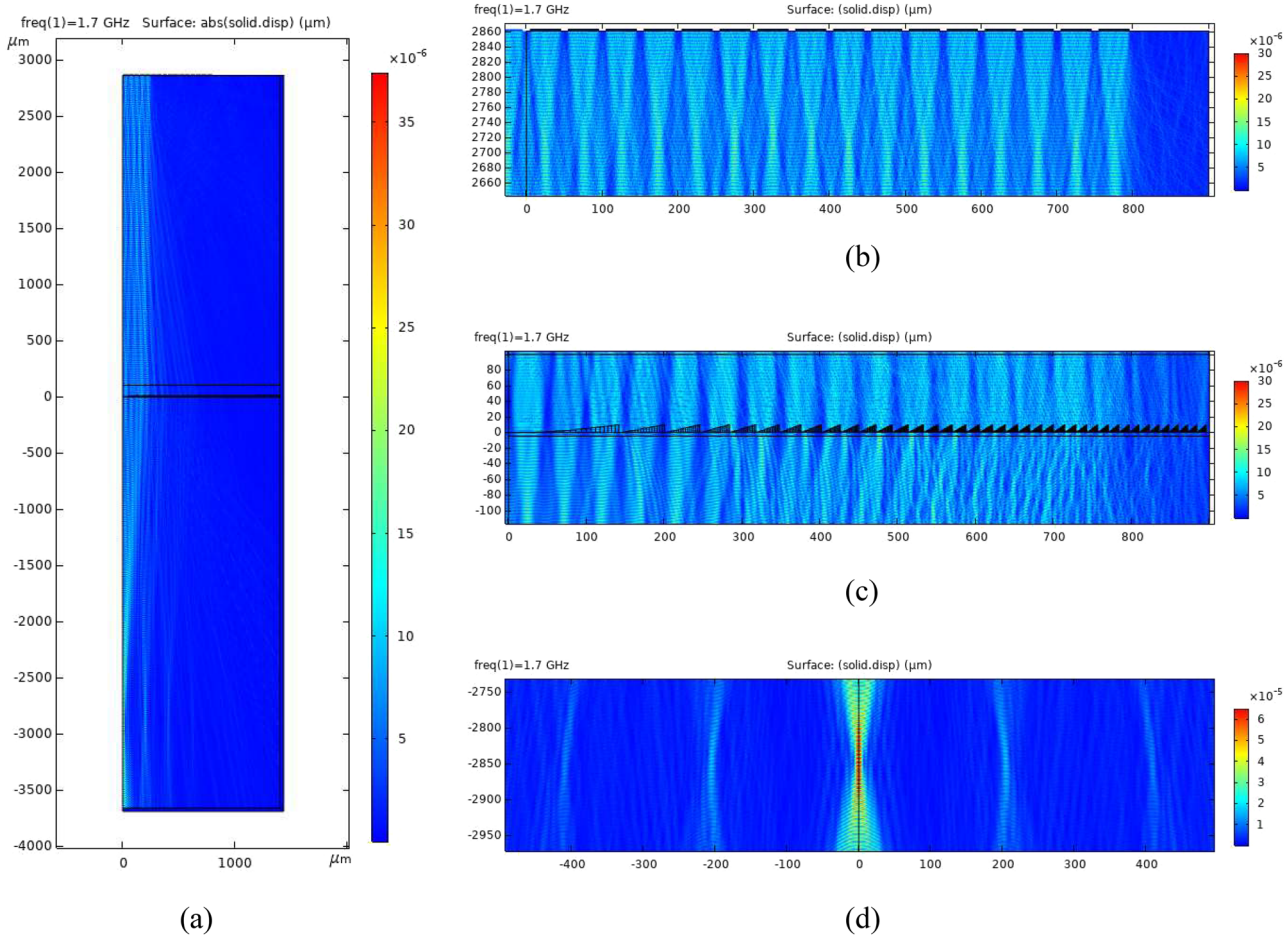
Case 1 which corresponds to the array of 10 piezoelectric transducers excited by 1-D voltage signals with 10 rectangular pulses. (**a**) The displacement magnitude solved by full-wave FEM simulation for a 10 transducer WFC block with 16-phase Fresnel lens. The displacement magnitude (**b**) near the transducer side by full-wave simulation, (**c**) around the 16-phase Fresnel lens zone, and (**d**) around the designed focal plane.

**Figure 6:**
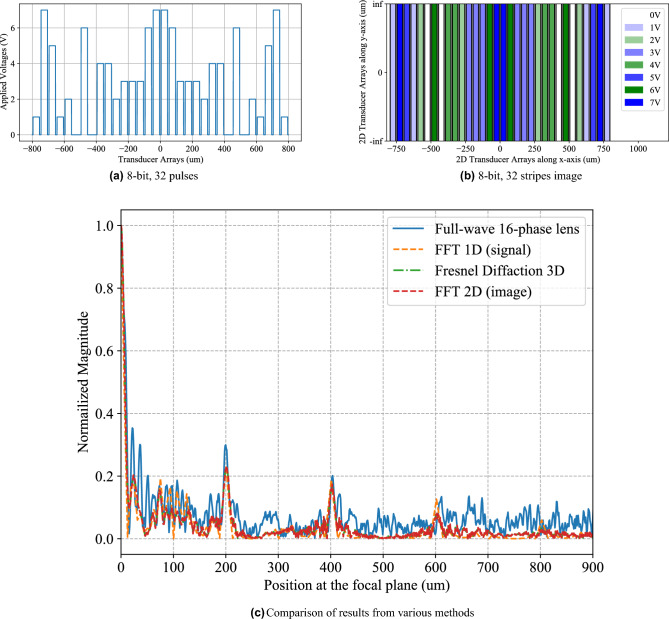
Case 2 which corresponds to the array of 32 piezoelectric transducers excited by (**a**) randomized 8-bit voltage signals. (**b**) Image of the input voltages at the input plane having 32 stripes represented by colored pixels. (**c**) Validation of the WFC system simulated by the full-wave FEM method, compared with digital FFT and Fresnel diffraction results using 16-phase Fresnel lens.

We simulated Case 1 which corresponds to a 1D array of 32 piezoelectric transducers with 10 transducers being activated by voltage signals (Fig. 3a). The 2D input voltages at the input plane are depicted by 10 stripes represented by blue and white pixels in Fig. 3b. The first order diffraction peak in the normalized magnitude of the Fourier transform computed by the full-wave simulation of a 16-phase Fresnel lens agrees well with the Fourier transforms computed by Fresnel diffraction and 1-D and 2-D digital methods (Fig. 3c). It is observed that higher diffraction orders do not match as well as the zeroth and first order peaks.

Performance degradation due to component imperfections can be modeled by the 2-phase, 4-phase and 8-phase Fresnel lenses which do not approximate the ideal phase profile of an ideal lens as well as the 16-phase Fresnel lens does. We performed full-wave FEM simulations for Case 1, using Fresnel lenses having different number of phase steps (Fig. 4). The Fourier transform computed by the 8-phase and 16-phase Fresnel lenses agree well with the digitally computed FFT. The Fourier transform computed by the 4-phase Fresnel lens exhibits slight deviations from the digitally computed FFT, while that computed by the 2-phase lens is drastically different from the digitally computed FFT. The 4-phase Fresnel lens provides a reasonable approximation to computing the Fourier transform. It must be noted that Fresnel lenses with more phase steps (e.g. $\ge$ 8-phase) are more difficult to fabricate than Fresnel lenses with fewer phase steps (e.g. $\le$ 4-phase).

We demonstrate for Case 1, the displacement magnitude in the Fused Silica medium, solved by full-wave FEM simulation for a 32 transducer WFC block in which only 10 transducers are activated, with a 16-phase Fresnel lens (Fig. 5a). The geometries in Fig. 5a–c are symmetric with respect to zero in the abscissa axis. The transducers array is at the y-coordinate of ${2860\,\upmu }m$ as shown in Fig. 5b. The displacement magnitude in Fused Silica near the transducer array is also shown. The 16-phase Fresnel lens along with the displacement magnitude is shown in Fig. 5c. From Fig. 5d, we observe that the Fresnel lens causes focusing at the focal plane with y-coordinate of ${-2860\,\upmu }$m.

To model a scenario which better resembles experimental conditions, we simulated Case 2 which corresponds to a 1D array of 32 piezoelectric transducers excited by randomized 8-bit voltage signals (Fig. 6a). The 2D input voltages at the input plane are depicted by 32 stripes represented by blue and white pixels in Fig. 6b. From positions from 0 to ${200\,\upmu }$m, we observe reasonable agreement between the normalized magnitude of the Fourier transform computed by the full-wave simulation of a 16-phase Fresnel lens, and the Fourier transforms computed by Fresnel diffraction and 1-D and 2-D digital methods (Fig. 6c). It is observed that higher diffraction orders do not match as well as the zeroth and first order peaks.

The average error and the $L^2$ norm error at the expected focal plane for 2, 4, 8, and 16-phase Fresnel lens are calculated by comparing the full-wave simulation with the 1-D and 2-D FFT results (Table 3). The normalized total displacement fields at the focal plane are simulated by full-wave FEM simulation and compared with the 1D normalized FFT results of Cases 1 and 2.

**Table 3:** The average error and the *L* norm error at the expected focal plane for 2, 4, 8, and 16-phase Fresnel lens for case 1: 10 uniform rectangular pulse signal and 10 uniform distributed 2D stripe image; and case 2: 32 randomized 8-bit pulse and 32 randomly distributed 2D stripe image.

Case	Lens phase	Compared with 1D FFT		Compared with 2D FFT	
		Average error	$L^1$ Norm error	Average error	$L^1$ Norm error
1	2	0.08393	228.376	0.08801	239.466
	4	0.04009	109.083	0.04262	115.981
	8	0.03649	99.2782	0.03799	103.380
	16	0.02283	62.1277	0.02397	65.2107
2	2	0.10403	283.066	0.10082	274.323
	4	0.06009	163.508	0.05675	154.418
	8	0.04616	125.601	0.04218	114.759
	16	0.04257	115.836	0.03844	104.587

The normalized total displacement fields at the focal plane are simulated by full-wave simulation and compared with the 1D normalized FFT result of case 1 and 2.

**Figure 7:**
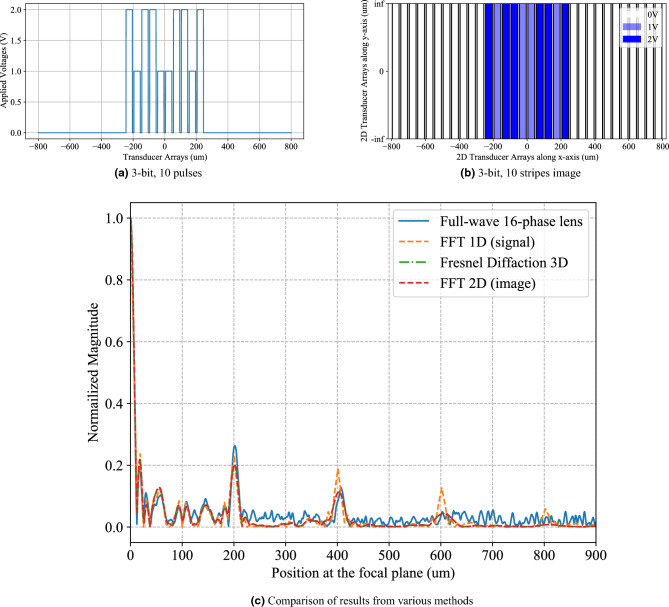
Case 3 which corresponds to the array of piezoelectric transducers excited by (**a**) randomized 3-bit voltage signals. (**b**) Image of the input voltages at the input plane having 10 stripes represented by colored pixels. (**c**) Validation of the WFC system simulated by the full-wave FEM method, compared with digital FFT and Fresnel diffraction results using 16-phase Fresnel lens.

We also demonstrate Case 3 which is an intermediate between Case 1 and Case 2. Case 3 corresponds to an array of 10 piezoelectric transducers excited by randomized 3-bit voltage signals (Fig. 7a). The input voltages at the input plane are depicted by 10 stripes represented by the shades of blue pixels in Fig. 7b. Similar to Cases 1 and 2, the higher diffraction orders do not match as well as the zero$^{th}$ and first order peaks (Fig. 7c).

**Figure 8:**
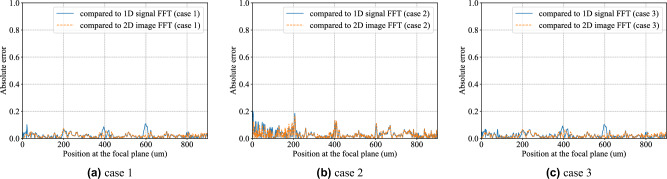
The absolute error compared with analytical 1D and 2D FFT results for case 1, 2, and 3.

**Figure 9:**
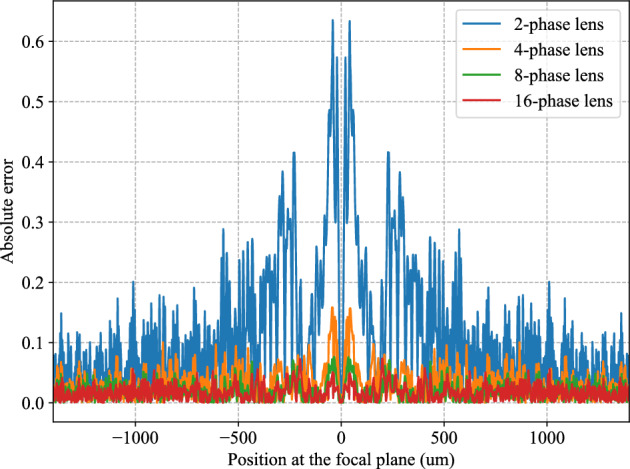
The absolute error compared with analytical 2D FFT results for case 1 using 2-, 4-, 8-, and 16-phase Fresnel lens.

In addition to the quantitative comparison (norm error for different multi-level phase Fresnel lens) given in Table 3, we also show the absolute error compared with analytical 1D and 2D FFT results for case 1, 2, and 3 in Fig.8. It is expected that case 1 has the minimum error compared to both 1D and 2D FFT results, and case 2 has more errors (within 0.2) as the input signals or image is the most complicated (random 8 bit input). Figure 9 shows the absolute error compared with analytical 2D FFT results for case 1 using 2-, 4-, 8-, and 16-phase Fresnel lens. It is observed that the absolute error reduces as the phase number increases. It is obvious 2-phase Fresnel lens is not suitable for the implementation of the GHz wavefront computing system due to the large error compared to analytical FFT results, while the accuray become better as the increase of the phase number of the Fresenel lens.

To summarize the errors shown by our comparisons for Cases 1, 2, and 3 in in Figs. 3c, 6c and 7c, respectively, we plot the absolute errors in Fig. 8 for the three cases. The absolute errors corresponding to Fig. 4 for the 2-phase, 4-phase, 8-phase, and 16-phase Fresnel lenses as compared to the CPU-computed 2D FFT are shown in Fig. 9.

## Discussion

The fabrication of the different components will lead to non-uniformities across the pixel array of piezoelectric transducers. The pixel array itself can have variations in pixel sizes due to lithography error. The lens pillars and radii will be affected by lithography errors. The bonding of the lens to the fused silica (Quartz) block may lead to bond-layer thickness variations. These thickness variations will produce variations in phase that will affect the phase shifts and the amplitudes of the acoustic waves received at the receiving piezoelectric sensor array.

Using the full-wave FEM simulation approach, we can have very accurate modeling, but it is time consuming. Using the Fresnel integral-based approach offers the possibility to scale up the simulation to very large array of transducers. However, the disadvantage of this approach is that there needs to be isotropic, lossless propagation medium. The use of the Fresnel diffraction integral also requires and a lens transfer function which needs to come from full-wave FEM simulations^16^.

In conclusion, we have presented the GHz ultrasonic wave piezoelectric instrumentation for Fourier transform computation, which we have demonstrated to perform reasonably accurate FT calculations. Our full-wave FEM simulations have showed the capabilities of the GHz Ultrasonic Wave Piezoelectric Instrumentation.

Our findings are significantly important. Performing FT computations faster than the Cooley–Tukey^8^ digital FFT algorithm, our instrumentation has the potential to meet the expanding need for such computations in uses like real-time video processing in self-driving automobiles. Our instrumentation has the potential to enhance the performance of wave-based analog computation devices to enable super-computers of the future.

## Data Availability

The data that support the findings of this study are available from the corresponding author upon reasonable request.
